# Three professions come together for an interdisciplinary approach to 3D printing: occupational therapy, biomedical engineering, and medical librarianship

**DOI:** 10.5195/jmla.2018.321

**Published:** 2018-07-01

**Authors:** Joan B. Wagner, Laurel Scheinfeld, Blanche Leeman, Keith Pardini, Jamie Saragossi, Katie Flood

**Affiliations:** Chief Librarian, Library, Touro College School of Health Sciences, Bay Shore, NY; Health Sciences Librarian, Stony Brook University, Stony Brook, NY; Clinical Assistant Professor, Stony Brook University, Stony Brook, NY; Librarian and Archivist, Long Island Room, Smithtown Public Library, Smithtown, NY; Head of Health Sciences Library, Stony Brook University, Stony Brook, NY; Library Assistant, Library, Touro College School of Health Sciences, Bay Shore, NY

## Abstract

**Background:**

Although many libraries have offered 3D printing as a service or available technology, there is a lack of information on course-integrated programs for 3D printing in which the library played a primary role. Therefore, librarians at the Touro College School of Health Sciences began exploring 3D printing for inclusion in the occupational and physical therapy curriculum.

**Case Presentation:**

The goal of this project was to educate occupational and physical therapy students and faculty about the potential applications of 3D printing in health care and provide hands-on experience, while increasing collaboration between librarians and faculty. Students’ tasks included designing and creating a 3D-printed assistive device as part of their course.

**Conclusion:**

Students were able to successfully print assistive devices, demonstrating the feasibility of 3D printing in a health sciences curriculum. Librarians involved with this project reached approximately 78 students and 200 other librarians and faculty members. 3D printing at Touro College continues to evolve and expand; the trial 3D printing course is being reviewed for formal adoption into the occupational therapy curriculum, and additional funding for 3D printing technologies is currently being allocated by Touro administration.

## BACKGROUND

Three-dimensional (3D) printing services in academic libraries have become more prevalent as the technology improves and becomes more accessible and financially sustainable. Many libraries, including academic libraries, have adopted the Makerspace model. In 2013, Bagley published a series of posts for the American Library Association defining the concept of makerspaces, in which 3D printers are common devices [1]. While she acknowledges that all makerspaces are different, she noted that a common focus is on creating and using new technologies to form communities of practice for creative problem-solving.

When a makerspace is provided with no specific instruction for 3D printer use, the devices are often left to collect dust. According to Radniecki and Klenke, the initial curiosity created by a 3D printer is good for the library, but simply printing trinkets does not exploit the technology to its full capabilities [2]. To provide a space for innovation and to use technology as a way to support institutional goals, it is necessary to provide more specific direction, training, and guidance in the use of the new technology [3].

Two EDUCAUSE reviews highlight 3D printing in academic libraries and encourage the involvement of librarians in creating pedagogy and developing a curriculum as a way to ensure the best use of 3D printers and to portray the library as a catalyst for innovation [4, 5]. In an online presentation from the National Network of Libraries of Medicine (NNLM), Anderson spoke about printing in health care, and many of her examples focused on the use of 3D printing for creating prosthetics [6]. For example, the organization e-NABLE works with a network of volunteers with 3D printers—including teachers, medical professionals, and engineering students—to provide upper limb assistive devices to those in need, such as children who outgrow traditional, expensive prosthetics [7]. Orthotics and other assistive devices can also be 3D-printed as highly customizable and low-cost alternatives for clients. For instance, in a case study by Day and Riley, 3D printing was used to provide an assistive device for a patient at a 56% lower cost than traditional products, which were not as effective as the 3D printed device [8].

After viewing the NNLM presentation on 3D printing [6], librarians at Touro College became interested in exploring the potential use of 3D printing in the curriculum. Touro College is a private, nonprofit institution serving 18,000 students worldwide. The Bay Shore, New York, campus is part of the School of Health Sciences, which offers degree programs in occupational therapy (OT) and physical therapy (PT), among others. Traditionally, OT and PT programs incorporate the creation and adaptation of medical assistive devices in the curriculum [9, 10].

Considering this existing framework along with the potential applications for 3D printing in these health care professions, Touro librarians identified the PT and OT departments as potential partners for a pilot curriculum that would introduce 3D printing technologies as a means for providing assistive devices to clients. It has been proposed that 3D printing skills provide additional opportunities for students who are preparing to enter the workforce [11]. Imparting this type of technological skill along with opportunities for critical thinking throughout the coursework was aligned with the programmatic goals at the School of Health Sciences. Therefore, the librarians developed a proposal for a pilot project and shared the proposal with the PT and OT departments, which immediately generated positive faculty responses and willingness to participate.

## STUDY PURPOSE

The primary purpose of this project was to introduce 3D printing to faculty and students using two pilot projects embedded in OT and PT coursework with the aims of providing hands-on experience with the new technology and increasing collaboration and academic engagement among librarians, students, and faculty at Touro College. A second goal was to share outcomes and lessons learned with the wider academic community and provide a project framework for other academic libraries.

## CASE PRESENTATION

During the planning phase, the authors contacted Patrick Colegrove, author of the EDUCAUSE review on 3D printing in academic libraries, to request information about the types of 3D printers purchased for libraries, the types of objects being printed, the cost of printers and supplies, and other information to help determine how to proceed with our project. Additionally, several brainstorming meetings were held between three Touro College librarians and three faculty members from the OT and PT departments.

A preliminary plan was developed that laid out the timeline, student project parameters, and roles of the involved parties. The project was initially funded by an NNLM Middle Atlantic Region Medical Library Project Award of $9,884 granted to Touro College Libraries in 2015, which allowed the library to purchase a Makerbot Replicator 3D printer, an extra filament extruder, and printer filament; to receive training in the use of the printer; and to obtain a service contract for the printer. In 2016, a School of Health Science Dean’s Research Award of $5,000 was granted for the purchase of a second 3D printer and a 3D scanner.

From our review of the literature and conversations with other academic librarians who were involved in 3D printing, we anticipated maintenance and technical issues; therefore, we enrolled in Makerbot’s support and warranty program and purchased an additional filament extruder. These turned out to be beneficial investments, as there were several malfunctions requiring shipment of parts and in-house repairs that were covered by the warranty. According to our records, we contacted Makerbot by phone or email thirteen times between November 2015 and December 2016 to help resolve printer problems, most of which were related to the extruders. During this time period, both printers needed to be returned and replaced once, while extruders were returned and replaced three times. The downtime due to repairs contributed to difficulty completing the required printing in the first year of the project, whereas the additional printer helped alleviate this problem in the second year.

During a two-day, in-person training course taught by a Makerbot representative, librarians realized that students and faculty would not have the skills necessary to design the 3D stereolithography (.STL) files with the level of detail required for the proposed projects, similar to previous observations made by health sciences librarians [12]. Therefore, we decided to explore alternative ideas for 3D modeling with other members of the 3D printing community. This led to a relationship between Touro College and EnableUC, a group of biomedical engineering students at the University of Cincinnati (UC), which was vital to the success of this project. This group had experience using computer-aided design (CAD) software and an interest in creating 3D .STL files for medical devices. Its members agreed to collaborate because they wanted to gain experience designing medical devices for actual patients. Thus, librarians served as intermediaries between Touro students and faculty and biomedical engineering students at UC.

Together with librarians, PT and OT faculty members developed course assignments for students to use the 3D printers to make assistive devices. The OT department introduced a new two-credit course on 3D printing, and the PT department incorporated one assignment into an existing three-credit course for a one-semester pilot project.

The new two-credit pilot course, “OT 686 Special Topics,” required students to identify a patient from their clinical rotations and create a prototype for a custom assistive device that would facilitate the patient’s ability to complete activities of daily living. Librarians created a VoiceThread presentation embedded in the course that introduced the 3D printing equipment that was available in the library and provided suggestions on creating successful assistive devices. Librarians also collaborated with the OT instructor by recommending additional videos and articles about 3D printing in health care. This 3D printing course has been offered three times to date.

In the first year, there were twenty-eight students, who produced twenty-eight prototypes, and print times for the assistive devices ranged from one to twenty hours. This volume of 3D objects was difficult to complete within our time frame. The curriculum was modified in subsequent years to have the students work in pairs, and the recommended dimensions of each device were restricted to under eight by four by three inches to reduce print times. During the second and third years, students produced eleven and fourteen prototypes, respectively, and the longest print time was seven hours.

Prototypes of the assistive devices were created with splinting material and other simple household materials. The prototypes were mailed by the library to UC to design the .STL files, and the completed .STL files were sent via email from UC to Touro librarians. Using Makerbot software, librarians displayed print previews of the devices to the OT students, which allowed a 360-degree view of objects to determine whether additional modifications were needed before printing. This preview also allowed librarians to use their 3D printing knowledge to determine appropriate print settings, such as the use of rafts or supports and the amount of infill. In order for the students to view their objects while they were being printed, librarians communicated with students to select a suitable time to schedule the printing.

Housing the 3D printer in the confines of the library allowed for extended print times and librarians supervision of the printing. The library team was able to keep the printer running at high volume, extending across different shifts and through the weekend. Despite several staffing changes, the library was able to maintain the continuity of the projects. Librarians supervised all aspects of printing, including loading filament and fixing filaments jams, and assisted with the post-prints, such as removing rafts or supports and smoothing surfaces.

Maintaining effective communication between various collaborators was imperative to the success of this project. Countless meetings, both in person and virtual, kept the project moving forward. Librarians created a spreadsheet in Google Drive to assist in tracking prototypes, files, and printed devices. This sheet proved essential in maintaining deadlines and keeping all parties informed (supplemental Appendix A).

**Figure 1:**
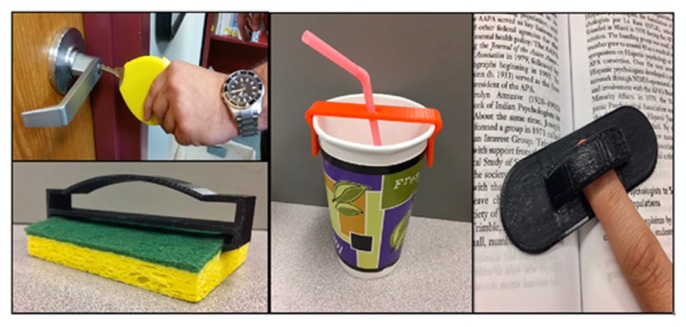
Examples of 3D-printed assistive devices * Top left: key turner; bottom left: sponge holder; center: straw stabilizer; right: book stabilizer.

In the first year, twenty-eight OT projects were created and twenty-four were printed; four files were never received from UC. Fifteen of the twenty-four projects were printed successfully as designed, four needed to be reprinted in a different size, and five needed adjustments that the UC designers were not able to complete before the end of the semester. In the end, eleven of the twenty-four printed projects were determined by the OT instructor to be useful assistive devices. In the second year, students were paired to create eleven prototypes, and all eleven files were received and printed. Nine of the eleven projects were determined to be useful devices. We are awaiting printing of the fourteen third-year files. A few examples of the completed projects include a key turner, sponge holder, straw stabilizer, and book stabilizer (Figure 1).

In the third year, a new element was added to the course: the design of a simple .STL file using a free, web-based beginner 3D modeling program called Tinkercad in order to introduce OT students to 3D design and give them an understanding of how the EnableUC engineering students created 3D .STL files of their prototypes. Uploading final projects to Thingiverse.com, Makerbot’s online 3D printing community, is also a requirement of the course. Uploading the projects promotes open access to the 3D-printed files and allows others to print the assistive devices. Librarians created training tutorials to help students accomplish these tasks.

For the PT pilot assignment, twenty-eight students who were enrolled in the “DPT 654 PT Interventions” course were required to design custom orthotics for the hand or foot. Students in groups of four or five designed a total of six orthotics to be 3D printed. Librarians gave a presentation to PT students on 3D printing in health care and introduced the 3D printing equipment available in the library. The files for custom orthotics proved more difficult to create than the OT assistive device files. The prototypes of the students’ designs had much more curvature, and UC engineering students were not able to create any acceptable .STL files. Librarians attempted to scan the prototypes using a 3D scanner or an iPad with the 123D Catch application to create image scans but had little success. Some problems that were encountered while scanning included glare from the splinting material, poor lighting in the library, and inability of the scanner to perceive depth. Unfortunately, none of the PT projects could be completed. This assignment was incorporated for one semester and has not been repeated.

## DISCUSSION

Overall, we believe this project has produced positive results, with the trial 3D printing OT course currently being reviewed for formal adoption into the OT curriculum. In addition, the dean of the Touro College School of Health Sciences has earmarked funds in 2018 for purchasing of a third 3D printer for the library. When embarking on this project, it was imperative that we received commitment from administration: if the project was successful, they would continue to financially support us after the initial grant monies were spent.

We also continue to believe there is a place for 3D printing in the PT curriculum. Similar to the difficulties faced at our institution, faculty at the University of Maryland reported barriers in printing certain types of PT devices due to fidelity and material that was available with standard 3D printers [13]. Touro librarians continue to search for alternative ways to create 3D printing files, with a specific focus on improving our scanning techniques in hopes of again working with the PT department for better results.

We also believe that educating students and faculty on the use of 3D printing in health care has been a worthwhile endeavor. OT students learned that 3D printing is a way to provide highly customizable, cost-effective, assistive technology solutions that can mitigate the “one size fits all” challenges seen with over-the-counter devices [14]. Students also have a new marketable job skill, and they will carry the experiential learning and creative problem-solving that they encountered during the course with them as they start their professional lives (supplemental Appendix B).

Along with the students benefiting from an introduction to a new technology, librarians also benefited from this experience by gaining a wide array of new skills, both through the formal training class taught by Makerbot and through reading, watching tutorials, and using trial and error. Operation of the 3D printers, use of software programs (i.e., 123D Design, Meshmixer, Tinkercad), iPad scanning, and scanning with the Makerbot Digitizer are now included in the librarians’ skill sets. The experience has allowed library staff to experiment with new technologies, which has increased their creativity and opportunities for future collaborative efforts. Library staff also gained a deeper understanding and appreciation of the OT and PT professions as well as the effort that therapists put into improving the lives of patients.

The 3D printers have improved the library’s visibility across all Touro College programs and campuses, not just those involved in the pilot courses. The central role of the library has been emphasized and recognized across the institution. The 3D printers increased traffic through the library and led to connections and conversations with students that might not have been made without the appeal of the printers. The library provided a venue for students and faculty to become more inclined to use technology and further develop relationships with library staff. Furthermore, interactions with students allowed the promotion of additional library services. The project has also increased the use of the embedded librarian program at Touro College, which was started to promote involvement of librarians at the course level. Embedded librarians’ roles have now expanded beyond traditional library instruction to include teaching about the use of new technology.

The library has also developed relationships that had not previously existed, primarily a partnership with UC and networks related to promotion of the 3D coursework. EnableUC continues to be an important partner in this project. Touro College made a small donation to the EnableUC group for their contribution to developing the .STL files. A future goal is to have more options for creating the .STL files, such as in-house design or additional local partners to ensure the continued viability of the project. Since the inception of the project, librarians have presented and shared information about the project at 7 events. Through conferences, meetings, and workshops, approximately 200 other librarians and faculty members have been reached. However, a much wider audience has been reached through social media, LibGuides, and news articles promoting the project, such as those in the Touro graduate student newsletter, National Libraries of Medicine blog, Touro library blog, community newspapers, and other Touro online newsletters. In particular, a 3D printing LibGuide was created to host and share information and media created in-house.

The library is now seen as a place for innovative technology. Representatives from other institutions including an OT department head at Touro University Nevada, library director at New York Medical College, and librarian at the Southampton campus of State University New York (SUNY)–Stony Brook have reached out to Touro librarians for information as they consider developing their own 3D printing projects. These connections will continue to help provide project frameworks. In addition, ideas for future collaboration are being discussed with SUNY–Stony Brook, and contacts have been made with Stony Brook biomedical engineering students with the possibility of creating a partnership similar to the one with EnableUC. One OT instructor at Touro has also reached out to the Stony Brook OT program to discuss the possibility of offering a graduate course in 3D printing at that campus.

The Touro College School of Health Sciences library is now increasingly positioned as a partner for faculty research projects and technology initiatives. The interdisciplinary relationships formed and the interest generated by the library 3D printers will facilitate future partnerships and projects.

## SUPPLEMENTAL FILES

Appendix A Google communication sheet

Appendix B Qualtrics survey
