# Supplementary material for: Three professions come together for an interdisciplinary approach to 3D printing: occupational therapy, biomedical engineering, and medical librarianship
Source: J Med Libr Assoc. 2018 Jul 1;106(3):370–6. doi: 10.5195/jmla.2018.321 (PMC6013144; doi:10.5195/jmla.2018.321)
Supplement: Appendix A [file jmla-106-370-s001.pdf]

JMLA Journal of the Medical Library Association

SC
Supplemental content to
J Med Libr Assoc. Jul;106(3):dx.doi.org/10.5195/jmla.2018.321
www.jmla.mlanet.org
© Wagner, Scheinfeld, Leeman, Pardini, Saragossi, Flood 2018

Three professions come together for an interdisciplinary approach to three-dimensional printing: occupational therapy, biomedical engineering, and medical librarianship

Joan B. Wagner, MLS; Laurel Scheinfeld, MLS; Blanche Leeman, MA, OTR/L, CHT; Keith Pardini, MLS; Jamie Saragossi, MLS; Katie Flood, BS

APPENDIX A

Google communication sheet

Item number	Scanned	Scan printed	Received file from UC Enable	File printed (student copy)	Picked up by student(s)	2nd copy printed for library	Project name	Drop off date	Estimated material use	Estimated cost of material (large spool of filament weighs 2 pounds/costs $48)	Print time	Notes on printing
1	9/20/16	9/27/16	11/7/16	11/9/16	11/21/16	11/14/16	Book stabilizer (helps hold the book open)	9/12/16	11.18 g (0.025 lb)	60 cents	50 mins	
2	9/29/16	10/26/16	11/3/16	11/7/16	11/10/16	11/29/16	Carton holder (to pour milk, cream)	9/14/16	73.22 g (0.16 Ib)	$3.84	6 hours + 20 mins	
3	9/29/16	10/27/16	11/3/16	11/10/16	11/17/16	11/14/16	Built-up toothpaste cap opener	9/14/16	23.44 g (0.052 lb)	$1.25	2 hours + 7 mins	
4	9/27/16	10/27/16	12/7/16	12/7/16	12/7/16	12/7/16	Typing aide (for someone with tremors)	9/14/16		67 cents	1 hour 38 m ins	
5	9/27/16 Chose the best option of multiple attempts	10/31/16	11/3/16	11/7/16	11/16/16	done (? date)	Bottle opener	9/19/16	21.76g (0.0481b)	$1.15	2 hours + 18 mins	

© Wagner, Scheinfeld, Leeman, Pardini, Saragossi, Flood 2018

Item number	Scanned	Scan printed	Received file from UC Enable	File printed (student copy)	Picked up by student(s)	2nd copy printed for library	Project name	Drop off date	Estimated material use	Estimated cost of material (large spool of filament weighs 2 pounds/costs $48)	Print time	Notes on printing
6	9/20/16	9/27/16	11/3/16	11/4/16	11/7/16	11/29/16	Sponge holder	9/19/16	17.88g (0.039 Ib)	94 cents	1 hour + 52 mins	
7	9/27/16 Chose the best 2 scans (7A&7B), both had their flaws	10/26/16	11/3/16	11/10/16	11/10/16	Done (?date), reprinted 12/14/16	Yogurt opener (pierces the foil)	9/21/16	146.84 g (0.324 lb)	$7.76	10 hours 25 mins	We reprinted laying down on new printer; 3 hours 30 min; 35.2g (0.078 lb); $1.87
8	9/27/16 Chose the best 2 scans (8A&8B), both had their flaws	10/20/16	12/7/16	12/7/16		12/8/16	Toothpaste and tooth brush aide (one on each end, to help someone who has tremors put toothpaste on the brush)	9/22/16	41.4g (0.091 lb)	$2.18	4 hours 36 mins	
9	9/27/16 Chose the best 2 scans (10A&10B), both had their flaws	10/28/16	12/9/16	12/12/16	12/13/16	12/12/16	Pants puller-upper	9/23/16	26.65g (0.059 lb)	$1.42	3 hours 50 mins	

Supplemental content to
J Med Libr Assoc. Jul;106(3):dx.doi.org/10.5195/jmla.2018.321
www.jmla.mlanet.org
© Wagner, Scheinfeld, Leeman, Pardini, Saragossi, Flood 2018

Item number	Scanned	Scan printed	Received file from UC Enable	File printed (student copy)	Picked up by student(s)	2nd copy printed for library	Project name	Drop off date	Estimated material use	Estimated cost of material (large spool of filament weighs 2 pounds/costs $48)	Print time	Notes on printing
10	9/29/16	10/21/16	11/17/16	11/22/16	11/28/16	11/21/16	Dog leash cuff	9/22/16	34.88g (0.077 lb)	$1.85	3 hours 58 mins	
11	9/27/16	9/27/16	11/7/16	11/9/16	11/9/16	11/30/16	Crochet aid (large piece)	9/23/16	13.38 g (0.029 lb)	70 cents	1 hour + 22 mins	
11a	9/27/16	9/27/16	11/7/16	11/9/16	11/9/16	11/30/16	Crochet aid (small piece)	9/23/16	2.33 g (0.005 lb)	12 cents	17 mins	
