# Supplementary material for: Three professions come together for an interdisciplinary approach to 3D printing: occupational therapy, biomedical engineering, and medical librarianship
Source: J Med Libr Assoc. 2018 Jul 1;106(3):370–6. doi: 10.5195/jmla.2018.321 (PMC6013144; doi:10.5195/jmla.2018.321)
Supplement: Appendix B [file jmla-106-370-s002.pdf]

JMLA Journal of the Medical Library Association

SC

Supplemental content to
J Med Libr Assoc. Jul;106(3):dx.doi.org/10.5195/jmla.2018.321
www.jmla.mlanet.org
© Wagner, Scheinfeld, Leeman, Pardini, Saragossi, Flood 2018

Three professions come together for an interdisciplinary approach to three-dimensional printing: occupational therapy, biomedical engineering, and medical librarianship

Joan B. Wagner, MLS; Laurel Scheinfeld, MLS; Blanche Leeman, MA, OTR/L, CHT; Keith Pardini, MLS; Jamie Saragossi, MLS; Katie Flood, BS

APPENDIX B

Qualtrics survey

Default question block

Do you agree that technology can improve patient care and treatment outcomes?

Strongly disagree	Disagree		Neither agree nor disagree	Agree	Strongly agree
0	1	2	3	4	5
Please select	●				

How familiar are you with 3D printing?

Never heard of it	Unfamiliar	Neither		Somewhat familiar	Have experience
0	1	2	3	4	5
Please select	●				

Are you interested in learning more about 3D printing?

Not interested	Somewhat interested		Not sure	Interested	Very interested
0	1	2	3	4	5
Please select	●				

Are you interested in creating items with a 3D printer?

Not interested	Somewhat interested		Not sure	Interested	Very interested
0	1	2	3	4	5
Please select	●				

How useful do you think 3D printing is in health care?

Very useless	Useless		Neutral	Useful	Very useful
0	1	2	3	4	5
Please select	●				

How likely are you to incorporate 3D printing into your future health care practice?

Very unlikely	Likely		Undecided	Likely	Very likely
0	1	2	3	4	5
Please select	●				

JMLA Journal of the Medical Library Association
SC
Supplemental content to
J Med Libr Assoc. Jul;106(3):dx.doi.org/10.5195/jmla.2018.321
www.jmla.mlanet.org
© Wagner, Scheinfeld, Leeman, Pardini, Saragossi, Flood 2018

For demographic purposes: Please identify gender
O Male
O Female
O Choose not to respond

For demographic purposes: Please identify age group
O 18–23
O 24–29
O 30+

What is your previous experience (if any) with patient care?
O Intern/volunteer with patients
O Paid position working with patients
O No previous patient experience
